# College students’ socioeconomic background and sleep during the first year of college

**DOI:** 10.1016/j.sleh.2025.06.002

**Published:** 2025-07-24

**Authors:** Sarah Rocha, Xochitl Arlene Smola, Ava Trimble, Luca Mc Donnell, Craig K. Enders, Andrew J. Fuligni

**Affiliations:** University of California, Los Angeles, Department of Psychology, Los Angeles, California, USA

**Keywords:** Sleep, Socioeconomic status, College students, Diary methods, Financial stress, Young adults

## Abstract

**Objective:**

Sleep problems can hinder adjustment to college, but limited work has evaluated whether students’ socioeconomic background is related to changes in sleep across the first year of college. The goal of the present study was to evaluate whether college-related financial stress, college generational status, and subjective social status were associated with differences in sleep across the first year of college.

**Methods:**

A total of *n* = 216 first-year college students (*M*Age = 18.1) at a public university were recruited for sleep quality assessment via the Pittsburgh Sleep Quality Index during the fall, winter, and spring academic quarters, and a subsample (*n =* 198) of these students participated in 14 days of sleep diary assessment at the beginning and end of the academic year.

**Results:**

Greater financial stress was significantly associated with worse sleep quality, more variable daily sleep duration, and greater difficulty waking up in the mornings. First-generation status was associated with poorer quality and more variable sleep, and worse subjective social status was linked to poor-quality sleep and greater difficulty awakening, but several of these associations were reduced after adjusting for ethnicity and gender. Mean-level socioeconomic indices did not interact with time, suggesting that between-person differences in sleep were consistent across the first academic year. Within-person increases in financial stress and subjective social status were associated with lower sleep variability.

**Conclusion:**

The study findings reveal significant socioeconomic-related differences in first-year college students’ sleep behavior. Intervention efforts to address college students’ sleep health may benefit from connecting low-socioeconomic status students with resources to facilitate adjustment to college.

## Introduction

Higher education can be a powerful tool for upward mobility,^1^ but students from socioeconomically disadvantaged backgrounds can face profound financial, academic, and social challenges when entering college^2^ that impact both well-being^3^ and likelihood of returning after the first year of college.^4,5^ One way that students from lower socioeconomic backgrounds may face issues with adjustment to college is through poor sleep. Sleep is a biological necessity^6^ with well-established links with learning and memory^7^ and mood.^8^ Prior epidemiologic research has documented that individuals of lower socioeconomic backgrounds are at heightened risk for a variety of sleep issues, including shorter duration, more variable sleep, and greater sleep complaints,^9,10^ with similar findings observed among adolescent populations.^11^ These links, however, have not been extensively studied during the first year of college—a period that can involve significant changes to youths’ sleep environments and sleep patterns.^12,13^ Sleep problems are highly prevalent among college students,^14^ and are known to contribute to both mental health struggles^15^ and diminished academic performance.^16^ Greater attention to the relation between students’ socioeconomic background and sleep during the first year of college could highlight one potential contributor to successful adjustment to college.

Importantly, socioeconomic status (SES) is a multifaceted construct that can be conceptualized through a variety of family resources, such as parents’ highest educational attainment, or family income and occupation.^17^ Socioeconomic indicators that are tailored to college contexts, such as first-generation college status (i.e., neither parent has obtained a bachelor’s degree) or difficulty affording college expenses, may be particularly likely to have implications for college student sleep. A substantial body of literature has documented that first-generation college students, many of whom are also from low-income families or are from minority racial and ethnic backgrounds that are under-represented in higher education,^18^ are more likely to report feelings of not belonging in college^19^ and experience psychological stress adjusting to a college environment.^2^ Psychosocial stressors such as these have known links with sleep difficulties^20–22^ as well as risk for internalizing disorders that are comorbid with sleep problems.^23–25^ Students with fewer financial resources to afford college expenses may be more likely to take on additional employment to pay for college costs, which can put a strain on time for sleep.^26^ Elements of physical sleep environment, such as insecure housing and overcrowding (e.g., multiple roommates sharing bedrooms) may also contribute to sleep problems for low-income students.^27,28^ Further, difficulties affording necessary expenses can lead to feelings of stress and anxiety that prevent the relaxed state needed for sleep onset.^29^

Importantly, these socioeconomic indicators, in addition to subjective perceptions of social status, have been linked to college students’ sleep in some preliminary research. For example, a survey study observed that students with greater self-reported difficulty affording college expenses and essential needs were more likely to self-report that they typically sleep < 7 hours per night.^30^ Another survey study observed that first-generation college students were more likely to self-report the quality of their sleep as “poor/fair” compared with “good/excellent.”^31^ Finally, a survey study observed that college students who perceived themselves as being of low social status were more likely to report typically sleeping less than 7-8 hours a night based on a categorical assessment of usual sleep duration.^32^ But findings across each of these studies are limited by their rudimentary assessments of sleep (i.e., categorical assessments of typical sleep duration and single-item assessments of overall quality). A rigorous evaluation of SES impacts on sleep patterns across the first year of college using daily sleep reports and well-validated sleep health questionnaires could provide stronger evidence for the role of socioeconomic resources on sleep during the first year of college. Additionally, prior work has evaluated these questions only at single timepoints; evaluating SES associations longitudinally across the first year of college can elucidate whether socioeconomic challenges contribute to increasing sleep problems across the academic year, furthering disparities in adjustment. These associations may be particularly important to examine across the first academic year, given that perceptions of social status may change as students interact with students of varying socioeconomic backgrounds^33^ and feelings of financial stress may fluctuate as students encounter novel challenges affording everyday college expenses.^34^

The present study proposed to address these associations through an exploratory, longitudinal investigation of SES and multiple components of sleep health across the first year of college in a sample of students at a large, public university in Los Angeles. Students were administered the well-validated Pittsburgh Sleep Quality Index (PSQI)^35^ at three timepoints across the academic year to obtain a comprehensive assessment of their overall sleep quality. Additionally, students’ sleep duration was assessed at the beginning and end of the academic year using self-reported daily sleep diaries over 14 days, a reliable and widely used tool for measuring sleep duration.^36^ These diaries were also used to measure the daily variability of students’ sleep duration and their perceived difficulty awakening. Consistent sleep schedules are a core component of proper sleep hygiene, and high sleep variability has been linked with poor health among youth and young adults over and above the duration of sleep.^37^

We selected three indicators of students’ socioeconomic background that can be easily self-reported by students and that capture different components of their college-related socioeconomic experiences (i.e., first-generation college status, college-related financial stress, and subjective social status [SSS]). We first examined whether these SES indices were associated with sleep at the start of the first year of college, and secondly, whether the SES indices predicted worsening sleep over time for lower-SES students compared with higher-SES students. Given the potential for feelings of financial stress and perceived SSS to fluctuate across the academic year, we further modeled whether within-person changes in financial stress and SSS were matched with within-person changes in sleep. Together, these longitudinal analyses could offer valuable directional evidence regarding the relation between SES and sleep across the first year of college.

## Methods

### Participants

A total of 216 college students (*M*_age_ = 18.1) entering their first year of study at a large public university in Los Angeles were recruited for a 1-year longitudinal assessment of sleep and health. Participants were recruited during the first week of instruction via classroom presentations, flyers, and email advertisements from the university registrar and departmental listservs. Students were eligible to participate in the study if they were 18-21 years of age and were entering their first year of study at the target university with no prior college experience (i.e., transfer students were not eligible to participate). Participants were 77.3% female, 19.4% male, and 3% identified as another gender or did not disclose gender identity (Table 1). The sample was ethnically diverse, with 50.0% of participants identifying as Asian/Pacific Islander, 27.8% as White, 19.9% as Hispanic/Latinx, and 2.3% identifying as another ethnicity. Consistent with the larger university student body, the majority (97%) of the sample lived in on-campus dormitories.

### Procedures

Data were collected during the second through eighth weeks of each of three, 10-week academic quarters (i.e., fall, winter, and spring). Each quarter, participants completed an online behavioral questionnaire regarding their socioeconomic background and subjective sleep quality. Retention rates were 89% (*n* = 192) and 83% (*n* = 179) in the second and third quarters.

A subsample of the initial 216 recruited participants chose to take part in the daily diary sleep assessment in the fall (*n* = 198) and spring quarters (*n* = 177). A link to an online sleep diary, which was part of a longer diary of daily experiences, was sent to participants each evening via text message. Participants were instructed to report their daily sleep behavior, including the time at which they woke up that morning, how difficult it was to get up, the time they went to bed the night prior, and the total amount that they slept in hours and minutes. Diaries reporting on sleep that occurred Friday through Saturday night were considered weekend sleep. Results for weekday sleep compared with weekend sleep are provided in the Supplement. Diaries were included in the analysis if received before noon the morning following the assigned diary day. To address potential recall bias associated with next-day report, sensitivity analyses removed diaries completed between 6:00 AM and 12:00 PM, which resulted in no changes to substantive findings (Supplement), and thus, these diaries were included in the main analysis. Compliance with diary procedures was high: those who participated in diary assessment completed on average 13 and 12 of the 14 daily diaries in the fall and spring, respectively.

Participants were compensated $30 cash after the first wave of data collection, $15 after the second wave, and $30 after the final wave. All procedures for this study were reviewed and approved by the UCLA Institutional Review Board.

### Measures

#### Socioeconomic background

##### First-generation college student status.

At study onset, participants reported the highest educational attainment of their parents or guardians, from 1 (some elementary education) to 11 (graduate or professional degree). Participants who reported no college degree completion for either parent/guardian were classified as first-generation college students (*n* = 46) and those who reported at least one parent completing a college degree classified as continuing-generation college students (*n* = 166). First-generation college student status was coded as a binary variable, such that 1 = first- generation college students and 0 = continuing-generation students. Four participants did not provide information on parents’ education.

##### College-related financial stress.

Stressors related to college expenses were assessed using a nine-item index^38^ at all three timepoints. The four-point Likert scale (endpoints “1 = never” and “4 = very often”) asked participants to report how often they experienced financial stressors, such as “worried about paying for college,” “chosen not to participate in an activity due to lack of money,” or “investigated withdrawing from college due to costs.” Cronbach’s alpha was > 0.81 across all study waves.

##### Subjective social status.

SSS was assessed via the MacArthur Scale of Subjective Social Status—Youth Version^39^ at all three timepoints. Participants selected the level they would place their family on a 10-rung ladder that represented where people stand relative to others in society, wherein the top of the ladder (10) reflects people with “the most money, the highest amount of schooling, and the jobs that bring the most respect,” and the bottom of the ladder (1) reflects people who “have the least money, little or no education, no job or jobs that no one wants or respects.”

#### Sleep

##### Subjective sleep quality.

Participants were asked to complete the PSQI at all three timepoints. The PSQI provides a comprehensive assessment of overall sleep quality by evaluating participants’ typical sleep over the past month across seven key components (i.e., duration, disturbance, latency, daily dysfunction due to sleepiness, efficiency, overall quality, and use of sleep medication) and has been validated for assessment among young adults^40^ and college students.^41^ Although the PSQI contains some overlapping information with the daily sleep diary (i.e., duration), the global score provides a reliable indicator of participants’ overall level of sleep disturbance,^35^ which is independently linked with health.^42^ Items on the PSQI are scored from 0-3 and the components are summed to obtain a global score of overall sleep quality. Global PSQI scores range from 0-21, with higher values indicating worse quality sleep, and scores > 5 indicating poor-quality or disturbed sleep. Cronbach’s alpha ranged from 0.56-0.65 across study waves.

##### Sleep duration.

Average nightly sleep duration in hours was estimated from the total sleep time reported in the 14 sleep diaries. In cases where a response to sleep time was missing (*n* = 5), nightly sleep duration was computed as the difference in hours from participants’ bedtime and waketime.

##### Variability.

Sleep variability was computed as the absolute value of the difference in hours between participants’ reported sleep duration each night and their average sleep duration of the 14-day period. Higher values reflect greater variability of daily sleep duration. Sensitivity analyses adjusted sleep variability estimates by participants’ sleep duration,^43^ which resulted in no substantive changes to study findings (Supplement).

##### Difficulty waking.

Perceived difficulty getting out of bed each morning was assessed across 14 daily sleep diaries, measured on a five-point scale, with endpoints 1 = “not difficult” and 5 = “very difficult.”

### Analysis plan

The present analyses evaluated whether college generation status, financial stress, and SSS were associated with students’ sleep outcomes (i.e., duration, variability, difficulty waking, and sleep quality) across the academic year. To this end, we ran a series of two-level, multilevel models in R statistical software (Version 4.4.0) that specified person-level (i.e., level-2) random intercepts to account for repeated measurements of sleep within participants. Model coefficients were estimated using a Bayesian latent variable estimation strategy with the rBlimp package (Keller, Version 0.1.31), which is adept at handling skewed variables (such as SES and sleep quality) and missing data due to attrition across time.^44^ The program’s Gibbs sampler estimated plausible parameter values using Markov chain Monte Carlo (MCMC) procedures with 10,000 iterations following a 1000-iteration burn-in period. The models utilized a default, non-informative prior distribution and model convergence was determined via the highest potential scale reduction (PSR) factor after each iteration. PSR per iteration was < 1.10 across models, indicating good convergence.^45^ Bayesian 95% credible intervals around each parameter provided probable ranges for SES effects on sleep outcomes, and intervals not including zero were interpreted as reflecting significant associations.

Missing values of predictor and outcome variables were assumed missing at random (MAR) and imputed via constructed distributions of possible missing data values for each MCMC iteration. Missing data rates of sleep indices ranged from 15.0%-15.4% and missingness of SES predictors ranged from 1.85%-9.88%. The final imputed samples consisted of *n* = 216 participants with 648 observations of PSQI-determined sleep quality and *n* = 198 participants with 5544 measurements of diary-reported sleep duration, variability, and difficulty waking.

The first series of models assessed the relations between the SES predictors and PSQI-determined sleep quality using a two-level nested structure, wherein level-1 reflected within-person variance in PSQI across all academic quarters, and level-2 reflected between-person variance in average PSQI (equations provided in the Supplement). First-generation status was determined at the fall quarter and was dummy coded as a between-person predictor (0 = continuing-generation, 1 = first-generation). A cross-level interaction between first-generation status and academic quarter (centered at the fall quarter) was added to the model to examine both the main effect of first-generation status on PSQI at the fall academic quarter and the influence of first-generation status on participants’ longitudinal change in PSQI across academic quarters.

Next, financial stress was measured at each academic quarter, and models examined the influence of both between-person differences in financial stress, as well as within-person fluctuations in financial stress across the academic year on PSQI. Mean-financial stress (centered at the grand-mean) was multiplied by academic quarter in a cross-level interaction. Additionally, within-person financial stress (centered within-person clusters) was multiplied by academic quarter in a “same-level” interaction. Thus, models estimated the main effects of both between-person and within-person differences in financial stress on PSQI, as well as their interactions with time, that is, academic quarter. These same steps were repeated for models evaluating the association of SSS with PSQI.

To examine SES associations with diary-reported sleep duration, variability, and difficulty waking, we again utilized a series of two-level multilevel models. Although the dataset contained three theoretical levels of nested variation (i.e., 14 diary days nested within 2 academic quarters nested within 198 participants), model ICCs indicated that minimal variation was explained by random intercepts at the level of the academic quarter (3.6%). Thus, the data were fit with a series of two-level models, wherein level-1 reflected within-person variance across 28 days of sleep diaries and level-2 reflected between-person variance in sleep diary outcomes. The main effects of all SES indices (i.e., first-generation status, mean-financial stress, within-financial stress, mean-SSS, and within-SSS) and interactions between SES indices and academic quarter were specified as above. Supplemental analyses evaluated these associations separately for weekdays compared with weekends (Supplement).

Follow-up models examined whether SES predictors were associated with sleep outcomes independent of other demographic variables. In these follow-up models, we repeated the above procedures additionally controlling for participants’ ethnicity (dummy coded with reference = White) and gender (dummy coded with reference = female).

## Results

### Pittsburgh Sleep Quality Index

**Fig. 1.**
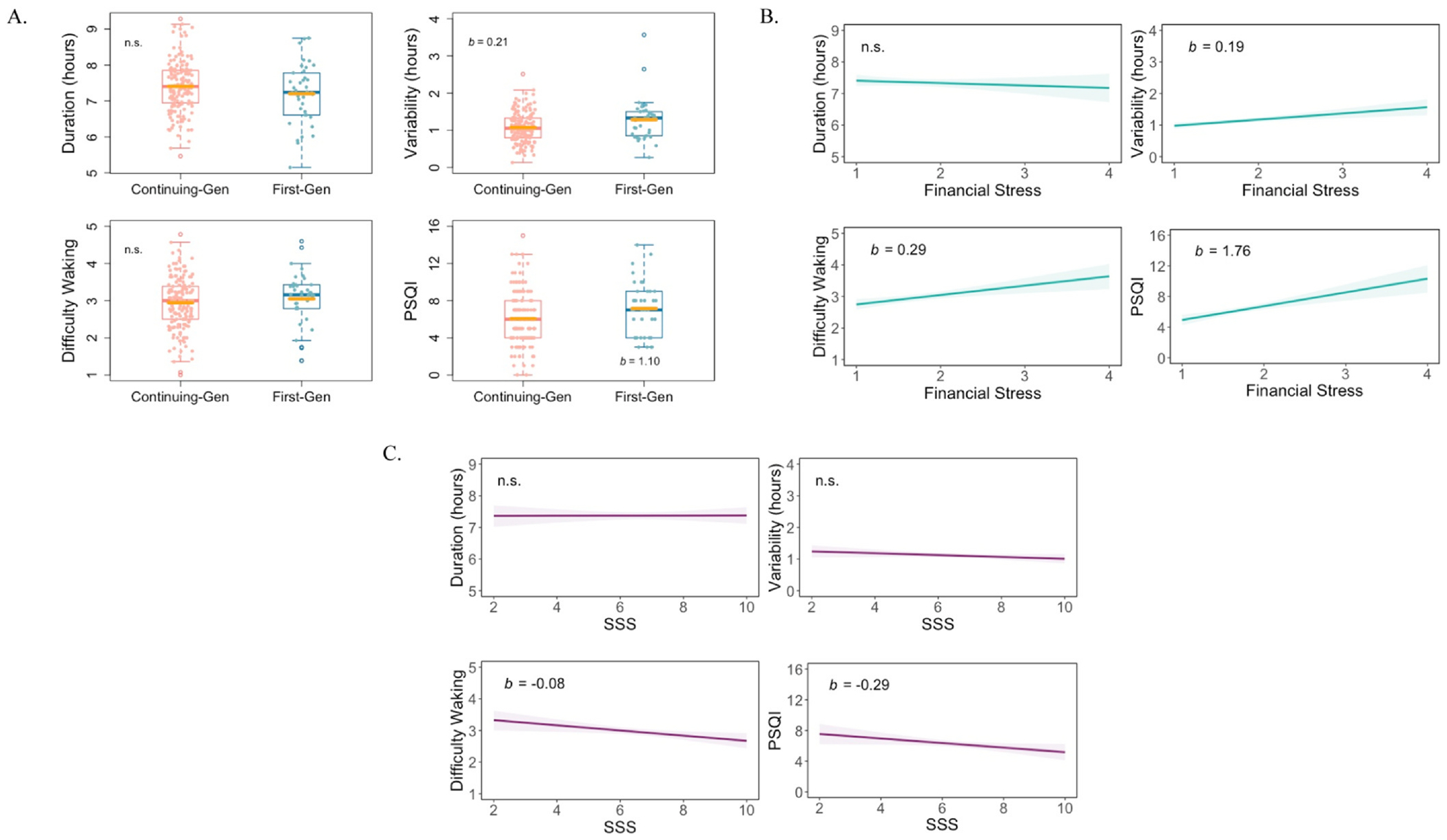
Visualization of SES associations with sleep duration, variability of duration, difficulty awakening, and PSQI. Panel A visualizes averaged sleep diary outcomes and PSQI scores at study onset for first-generation college students compared with continuing-generation students in box-and-whisker plots. Means are denoted with orange lines and betas are reported for significant mean differences. Nonsignificant associations are denoted as “n.s.” Panels B and C display multilevel associations of mean-level college-related financial stress and SSS with sleep outcomes, respectively. Shaded regions reflect 95% credible intervals around the multilevel estimates. PSQI, Pittsburgh Sleep Quality Index; SES, socioeconomic status; SSS, subjective social status

As shown in Table 2, first-generation college students had approximately one-point higher PSQI scores—indicating worse sleep quality—than continuing-generation students (*b* = 1.10 [0.11, 2.07]) at study onset. This association became nonsignificant after controlling for participants’ ethnicity and gender (see Table 3). Male participants had lower PSQI scores compared with female participants (Table 3). Participants reporting higher college-related financial stress also had significantly higher PSQI scores (*b* = 1.76 [0.97, 2.56]; Fig. 1), and this remained the case even after accounting for demographic variables (Table 3). Similarly, those reporting higher (better) SSS had lower (better) PSQI scores (*b* = −0.29 [−0.58, −0.01]), though only in models not controlling for gender and ethnicity. Within-person fluctuations in financial stress and SSS were not associated with sleep quality. Finally, none of the SES indices were associated with longitudinal change in PSQI scores across the academic year.

### Sleep duration

Between-person SES predictors were not significantly associated with students’ sleep duration at study onset, nor did they predict change in students’ sleep duration across time (see Table 2). After adjusting for demographic variables, within-person increases in financial stress were associated with longer sleep duration (*b* = 0.67 [0.04, 1.30]). Latinx and Asian students had shorter sleep duration compared with White students, and there were no differences in duration by gender (Table 3).

### Sleep variability

First-generation students had significantly greater sleep variability (*b* = 0.21 [0.05, 0.37]) compared with continuing-generation students, though these links became nonsignificant after accounting for gender and ethnicity (Table 3). Next, higher mean-financial stress (i.e., a “between-person” difference) was significantly associated with greater sleep variability (*b* = 0.19 [0.08, 0.29]), while within-person increases in financial stress above one’s mean were associated with lower sleep variability (*b* = −0.42 [−0.80, −0.07]); these estimates remained significant even after accounting for students’ gender, ethnicity (Table 3). Between-person differences in SSS were not significantly associated with sleep variability (Table 2), but within-person increases in SSS were associated with lower sleep variability (*b* = −0.13 [−0.26, −0.01]), even after adjusting for gender and ethnicity. None of the SES variables significantly interacted with academic quarter.

### Difficulty waking

First-generation status was not significantly associated with difficulty waking up in the morning (Table 2). Students with higher mean-financial stress reported greater difficulty waking in the morning (*b* = 0.29 [0.10, 0.47]), while those with higher (better) average SSS reported less difficulty waking (*b* = −0.08 [−0.15, −0.02]). These associations remained true after accounting for ethnicity and gender. Fluctuations in students’ financial stress and SSS were not linked with difficulty waking up at the start of the year (Table 2), but there was a significant interaction between within-person SSS and academic quarter (*b* = −0.43 [−0.86, −0.03]), such that within-person increases in SSS were associated with less difficulty waking in the spring academic quarter (*b* = −0.26 [−0.48, −0.04]). Male participants had less difficulty waking, while those of another gender reported greater difficulties waking (Table 3).

### Weekends vs. weekdays

SES-sleep associations did not vary when evaluating weekdays separately from weekends (Supplement). In relation to weekend sleep, financial stress remained significantly associated with greater perceived difficulty waking (Supplement), but other SES indices were no longer associated with difficulty waking, nor with sleep variability (Supplement).

## Discussion

The present study evaluated whether students’ socioeconomic background was associated with sleep health across the first year of college. We observed that higher average financial stress over college expenses was related to poorer PSQI-determined sleep quality, greater day-to-day variability in diary-measured sleep duration, and greater perceived difficulty waking up in the mornings across the first year of college. First-generation status was also associated with poorer quality and more variable sleep, but these links were attenuated after accounting for students’ gender and ethnic background. Perceived social status was linked with less difficulty waking and higher-quality sleep, though the latter finding did not hold after adjusting for their gender and ethnic background. Models suggested that between-person SES-sleep associations were stable across the year; we did not find evidence that between-person SES characteristics were related to longitudinal differences in sleep across the academic year. Regarding within-person fluctuations in SES, we found that within-person increases in financial stress were associated with lower sleep variability, while within-person increases in SSS were associated with lower sleep variability in the fall and less difficulty waking in the spring quarter.

Among SES predictors, the study findings particularly highlighted financial stress over college expenses as a predictor of sleep quality and daily sleep behavior (i.e., variability, difficulty waking), but not the total duration of students’ sleep. The latter finding is somewhat inconsistent with prior epidemiologic studies that observe shorter sleep duration among lower-SES individuals,^9^ but these studies tend to focus on factors such as income and educational attainment that may not capture the impact of college-specific financial demands. College sleep-wake routines also have notable differences from other populations given students’ flexible course schedules.^13^ Irregular start times could more easily allow students to “make up” for short nights of sleep by sleeping in on subsequent days. Instead of shortening total duration, financial difficulties instead may operate on sleep health by increasing the irregularity of sleeping patterns. In line with this, we observed that students with higher financial stress (and those identifying as first-generation college students in unadjusted models) had greater day-to-day variability in their sleep duration. Regardless of total duration, a more variable pattern of sleep duration is concerning given links with poor physical and mental health.^46,47^ Significant deviations in the timing of one’s bedtime and waketime can alter circadian processes that induce feelings of wakefulness or sleepiness,^48^ which may be one reason why higher sleep variability was paired with greater perceived difficulty waking up.

One of several potential pathways by which low SES may be linked with sleep could be students’ need to take on additional employment to pay for college expenses, which has been previously shown to place strains on sleep.^26^ Elements of physical sleep environment, such as insecure housing and overcrowding (e.g., multiple roommates sharing bedrooms) may also contribute to sleep problems for students with fewer financial resources.^27,28^ Difficulties affording necessary expenses may induce feelings of stress and anxiety that could prevent the relaxed state needed for sleep onset.^29^ Finally, psychosocial stressors associated with the start of college may play a role in sleep disruptions. For example, prior work has found that low-SES students are more likely to feel out of place or perceive lower social evaluation by others when navigating a high-SES college environment.^2,49^ Social stress can negatively impact sleep^21,22^ and increase risk for poor mental well-being.^24^ Stressors such as these may be particularly acute for low-SES students who identify as first-generation college students and those with low perceived status.^19,49^ Although some SES-sleep associations (e.g., those related to first-generation status) did not hold after accounting for ethnicity and gender, it can be challenging to assess these variables separately when there is a substantial overlap in marginalized identities (e.g., 87% of first-generation college students in the present sample identified as either Latinx or Asian, and 89% identified as female). Prior literature suggests that female college students, as well as Latinx and Asian students, may have poorer quality sleep compared with White college students.^50^ In the present study, we similarly observed that female students had worse overall sleep quality and Latinx and Asian students had shorter sleep duration. Thus, it is likely that some of the effects of first-generation status may be related to other aspects of students’ gender and ethnic identity. Additional research utilizing larger, within-group analyses of SES and sleep is needed to examine whether these effects exist independent of students’ gender and ethnic background.

Interestingly, between-person SES predictors did not interact with time, indicating that sleep problems were consistently higher among low-SES students, rather than developing over the course of the academic year. While this finding limits directional inferences about the relationship between SES and college sleep, it provides important correlational evidence of socioeconomic disparities in college student sleep that warrants future research in larger samples across multiple campus environments. The within-person SES-sleep findings provided some evidence of a directional relation between SES fluctuations and sleep during the first year of college, as within- person increases in SSS were linked with lower sleep variability and less difficulty waking. Surprisingly, within-person increases in financial stress were also linked with declines in sleep variability. Potentially, this latter finding could reflect declines in work hours or loss of employment that free up time for sleep but also heighten financial concerns. It is possible that additional sleep changes may emerge later in students’ college careers (e.g., second through fourth year), when students increasingly balance heavy course loads and may no longer qualify for on-campus university housing. Future longitudinal research may clarify the role of SES on sleep trajectories by measuring SES and sleep over multiple timepoints across the 4 years of college. Compared to our narrow assessment of the first year of college, such data would also provide greater variance for assessing the impact of within-person fluctuations in SES students’ sleep. For example, examining changes in students’ finances or changes in institutional financial aid throughout the 4 years of college could offer a higher-powered evaluation of how SES fluctuations shape sleep across college.

Limitations of the present work include the potential for recall bias when using an evening sleep diary to evaluate prior night’s sleep and same days’ difficulty waking, as participants may have not remembered the exact duration of their sleep and ease of awakening. Additionally, we did not exclude participants based on prior diagnosis of sleep or psychiatric disorders, use of sleep medications, caffeinated beverages, or substance use, which could have influenced sleep results. Next, the gender and ethnic distribution of the sample was reflective of the demographic composition of the University of California, Los Angeles, however, the present sample had a slightly higher proportion of women and Asian participants, indicating potential for self-selection bias. Findings from the present study may not generalize to other gender and racial/ethnic groups, particularly African Americans, who on average experience both higher burdens of financial strain^51^ and under-representation in higher education.^52^ Additional work evaluating these links among samples of African American college students is warranted. Finally, findings are correlational in nature and cannot determine causal relations between SES and sleep, further, findings do not indicate whether SES indices (e.g., first-generation status and financial stress) operate independently of each other on sleep.

Despite these limitations, our findings highlight potential targets for intervention to address disparities in sleep across the first year of college. Insufficient financial support is cited as a major factor behind college attrition.^53^ Alleviating students’ financial concerns through greater financial aid and connecting first-generation college students with resources to support adjustment to college could be among a few ways universities might address disparities in college students’ sleep. Additionally, intervening directly to improve sleep—such as promoting proper sleep hygiene^54^—could be a low-cost avenue to promote sleep health for first-year students. The high burden of sleep problems in the overall sample was also concerning (e.g., 50% of participants exhibited sleep disturbance as evidenced by PSQI score > 5) and is consistent with patterns observed among other colleges and universities following the COVID-19 pandemic.^55^ Investing in students’ sleep health—particularly for socioeconomically disadvantaged students who may be at higher risk for poor sleep—may be a key pathway to the promotion of both well-being and adjustment to college.

## Conclusion

Students from lower socioeconomic backgrounds can face heightened challenges during the first year of college that may become manifested in sleep difficulties. This study offered a novel investigation of the association of college generational status, college-related financial stress, and perceived social status on multiple dimensions of sleep health across the first year of college. The study findings indicated that lower-SES indicators were associated with poor sleep quality, higher sleep variability, and difficulties with awakening in the morning. Results from this study further emphasize the importance of assessing socioeconomic measures that capture students’ college-specific socioeconomic experiences, such as financial stress over college expenses. Future research evaluating the potential long-term consequences of college-related financial difficulties on student sleep health across diverse samples is warranted. Intervention efforts to address disparities in college sleep health may benefit from targeting first-generation college students and other lower-SES students for sleep hygiene programs in addition to connecting students with resources for college-related financial support.

## Supplementary Material

1

## Figures and Tables

**Table 1:** Descriptive statistics of main variables of interest at each study timepoint

Measure	Fall		Winter		Spring	
	*n* = 216		*n* = 192		*n* = 179	
	*n* (%)	Mean (SD)	*n* (%)	Mean (SD)	*n* (%)	Mean (SD)
SES						
First generation	46 (22)	-	35 (18)	-	33 (18)	-
Financial stress	-	1.77 (0.59)	-	1.74 (0.55)	-	1.68 (0.57)
SSS	-	6.42 (1.80)	-	6.46 (1.64)	-	6.60 (1.57)
Demographics						
Female gender	167 (77)	-	150 (78)	-	141 (79)	-
Male gender	42 (19)	-	37 (19)	-	33 (18)	-
Another gender	7 (3)	-	5 (3)	-	5 (3)	-
Asian ethnicity	108 (50)	-	99 (52)	-	93 (52)	-
Latinx ethnicity	43 (20)	-	35 (18)	-	33 (18)	-
White ethnicity	60 (28)	-	55 (29)	-	51 (28)	-
Another ethnicity	5 (2)	-	3 (2)	-	2 (1)	-
Sleep						
PSQI	-	6.31 (2.83)	-	5.85 (2.88)	-	6.10 (2.97)
Average sleep duration	-	7.37 (0.73)	-	-	-	7.37 (0.92)
Average sleep variability	-	1.11 (0.45)	-	-	-	1.08 (0.53)
Average difficulty waking	-	2.96 (0.72)	-	-	-	3.06 (0.78)

Abbreviations: PSQI, Pittsburgh Sleep Quality Index; SD, standard deviation; SES, socioeconomic status; SSS, subjective social status.

*n* reflects participants who provided at least some SES or sleep-related data at a given wave. Percentages (%) have been rounded to the nearest whole number and may not sum to 100.

**Table 2:** Unadjusted multilevel associations of SES predictors with average and longitudinal sleep outcomes

		I. PSQI			II. Difficulty waking			III. Duration			IV. Variability		
Effect-level	SES index	*B*	2.5%	97.5%	*B*	2.5%	97.5%	*B*	2.5%	97.5%	*B*	2.5%	97.5%
Between-person (L-2)	First-generation	**1.10**	0.11	2.07	0.10	−0.17	0.37	−0.19	−0.47	0.09	**0.21**	0.05	0.37
	First-generation*Quarter	−0.36	−0.86	0.15	−0.07	−0.24	0.09	0.13	−0.11	0.37	−0.05	−0.20	0.09
Between-person (L-2)	Financial stress	**1.76**	0.97	2.56	**0.29**	0.12	0.47	−0.09	−0.28	0.11	**0.19**	0.08	0.30
	Financial stress*Quarter	−0.14	−0.56	0.30	0.04	−0.07	0.16	−0.10	−0.28	0.08	0.02	−0.08	0.13
Within-person (L-1)	Financial stress	−0.92	−2.32	0.53	−0.42	−1.09	0.22	0.62	−0.05	1.27	−**0.42**	−0.80	−0.07
	Financial stress*Quarter	0.32	−1.05	1.69	0.79	−0.48	2.08	−0.96	−2.20	0.28	0.47	−0.19	1.16
Between-person (L-2)	SSS	−**0.29**	−0.58	−0.01	−**0.08**	−0.15	−0.02	0.002	−0.07	0.07	−0.03	−0.07	0.01
	SSS*Quarter	0.01	−0.14	0.16	0.03	−0.01	0.07	0.001	−0.06	0.06	−0.01	−0.05	0.03
Within-person (L-1)	SSS	0.10	−0.38	0.57	0.18	−0.03	0.40	0.17	−0.03	0.38	−**0.15**	−0.26	−0.03
	SSS*Quarter	−0.24	−0.66	0.20	−**0.43**	−0.86	−0.03	−0.27	−0.64	0.11	0.18	−0.04	0.39

Abbreviations: L-1, level-1; L-2, level-2; PSQI, Pittsburgh Sleep Quality Index; SSS, subjective social status.

The table reports unstandardized Bayesian point estimates and 95% credible intervals around these estimates for each sleep outcome regressed by between-person socioeconomic status (SES) predictors (L-2) and within-person SES predictors (L-1). Note that first-generation status contains only between-person variation. L-1 = Level-1; L-2 = Level-2; SSS = Subjective Social Status; PSQI = Pittsburgh Sleep Quality Index.

Bayesian estimates are bolded in the table if the corresponding credible interval does not include zero.

**Table 3:** Multilevel SES associations with sleep outcomes after adjusting for participants’ demographic characteristics

		I. PSQI			II. Difficulty waking			III. Duration			IV. Variability		
SES effect-level	Predictor	*B*	2.5%	97.5%	*B*	2.5%	97.5%	*B*	2.5%	97.5%	*B*	2.5%	97.5%
Between-person (L-2)	First-generation	0.70	−0.48	1.87	0.06	−0.23	0.33	0.01	−0.30	0.31	0.14	−0.04	0.32
	First-generation * Quarter	−0.34	−0.86	0.18	−0.06	−0.23	0.10	0.14	−0.10	0.38	−0.04	−0.19	0.10
	Male gender	**−1.06**	−1.93	−0.19	−0.24	−0.49	0.01	0.16	−0.11	0.43	0.01	−0.14	0.16
	Another gender	−0.84	−3.15	1.45	**0.66**	0.06	1.28	0.03	−0.61	0.67	−0.06	−0.41	0.31
	Asian ethnicity	0.35	−0.45	1.12	0.17	−0.07	0.40	**−0.29**	−0.54	−0.06	−0.03	−0.17	0.11
	Latinx ethnicity	0.73	−0.48	1.91	0.16	−0.17	0.47	**−0.50**	−0.86	−0.17	0.13	−0.07	0.32
	Another ethnicity	0.94	−1.98	3.85	0.12	−0.78	0.91	0.06	−0.86	0.93	**0.54**	0.01	1.03
Between-person (L-2)	Financial stress (L-2)	**1.77**	0.99	2.57	**0.30**	0.12	0.48	−0.01	−0.21	0.20	**0.16**	0.04	0.28
	Financial stress (L-2) * Quarter	−0.16	−0.58	0.26	0.04	−0.07	0.15	−0.09	−0.27	0.08	0.02	−0.09	0.12
Within-person (L-1)	Financial stress (L-1)	−1.09	−2.52	0.33	−0.37	−1.06	0.29	**0.67**	0.04	1.30	**−0.44**	−0.83	−0.07
	Financial stress (L-1) * Quarter	0.49	−0.88	1.88	0.69	−0.60	2.02	−1.09	−2.27	0.09	0.50	−0.19	1.21
	Male gender	**−1.12**	−2.01	−0.25	**−0.25**	−0.50	−0.01	0.17	−0.10	0.42	< 0.001	−0.14	0.14
	Another gender	−1.01	−3.12	1.10	**0.58**	0.03	1.17	0.07	−0.56	0.71	−0.12	−0.47	0.24
	Asian ethnicity	0.23	−0.53	1.02	0.15	−0.06	0.38	**−0.30**	−0.53	−0.06	−0.03	−0.16	0.11
	Latinx ethnicity	0.12	−0.93	1.15	0.02	−0.26	0.32	**−0.43**	−0.75	−0.12	0.12	−0.05	0.30
	Another ethnicity	0.29	−2.48	3.11	−0.06	−0.81	0.76	0.02	−0.81	0.90	0.45	−0.02	0.95
Between-person (L-2)	SSS (L-2)	−0.25	−0.58	0.08	**−0.08**	−0.15	−0.003	−0.05	−0.13	0.02	−0.01	−0.05	0.04
	SSS (L-2) * Quarter	0.002	−0.15	0.15	0.03	−0.01	0.07	< 0.001	−0.06	0.06	−0.01	−0.05	0.03
Within-person (L-1)	SSS (L-1)	0.08	−0.39	0.56	0.22	−0.002	0.42	0.17	−0.03	0.38	**−0.13**	−0.26	−0.01
	SSS (L-1) * Quarter	−0.21	−0.63	0.22	**−0.51**	−0.91	−0.08	−0.26	−0.64	0.13	0.15	−0.09	0.38
	Male gender	**−1.16**	−2.07	−0.26	**−0.24**	−0.50	0.004	0.15	−0.11	0.41	0.00	−0.15	0.14
	Another gender	−0.88	−3.06	1.32	**0.69**	0.12	1.30	0.03	−0.59	0.68	−0.10	−0.46	0.27
	Asian ethnicity	0.21	−0.60	1.04	0.16	−0.05	0.40	**−0.31**	−0.54	−0.06	−0.04	−0.18	0.10
	Latinx ethnicity	0.35	−0.91	1.59	0.08	−0.26	0.40	**−0.56**	−0.93	−0.22	0.16	−0.05	0.36
	Another ethnicity	0.67	−2.20	3.61	0.15	−0.63	1.00	0.05	−0.79	0.94	0.45	−0.04	0.95

Abbreviations: L-1, level-1; L-2, level-2; PSQI, Pittsburgh Sleep Quality Index; Reference = White, female.

The table reports unstandardized Bayesian point estimates and 95% credible intervals around these estimates for each sleep outcome regressed by socioeconomic status (SES) predictors and demographic control variables. Note that first-generation status contains only between-person variation.

Bayesian estimates are bolded in the table if the corresponding credible interval does not include zero.

## Appendix A. Supporting information

Supplementary data associated with this article can be found in the online version at doi:10.1016/j.sleh.2025.06.002.
