# Supplementary material for: College students’ socioeconomic background and sleep during the first year of college
Source: Sleep Health. Author manuscript; Available in PMC 2026 Jun 19. (PMC13281785; doi:10.1016/j.sleh.2025.06.002)
Supplement: 1 [file NIHMS2178887-supplement-1.docx]

**Supplement**

Supplemental Table 1. Multilevel SES associations after removing sleep diaries received in the morning

|  |  | Difficulty Waking | | |  | Duration | | |  | Variability | | |
| --- | --- | --- | --- | --- | --- | --- | --- | --- | --- | --- | --- | --- |
|  |  | Estimate | 2.5% | 97.5% |  | Estimate | 2.5% | 97.5% |  | Estimate | 2.5% | 97.5% |
|  |  |  |  |  |  |  |  |  |  |  |  |  |
| Between-Person (L-2) | First-Generation | 0.14 | -0.14 | 0.41 |  | -0.10 | -0.39 | 0.20 |  | **0.21** | 0.04 | 0.38 |
|  | First-Generation*Quarter | -0.09 | -0.29 | 0.11 |  | 0.14 | -0.15 | 0.42 |  | -0.04 | -0.21 | 0.13 |
|  |  |  |  |  |  |  |  |  |  |  |  |  |
| Between-Person (L-2) | Financial Stress | **0.31** | 0.13 | 0.50 |  | -0.04 | -0.24 | 0.18 |  | **0.17** | 0.05 | 0.29 |
|  | Financial Stress*Quarter | -0.02 | -0.15 | 0.11 |  | -0.03 | -0.23 | 0.16 |  | 0.04 | -0.08 | 0.15 |
|  |  |  |  |  |  |  |  |  |  |  |  |  |
| Within-person (L-1) | Financial Stress | -0.28 | -0.94 | 0.37 |  | **0.76** | 0.10 | 1.40 |  | **-0.57** | -0.96 | -0.19 |
|  | Financial Stress*Quarter | 0.57 | -0.70 | 1.84 |  | -1.11 | -2.30 | 0.15 |  | **0.78** | 0.09 | 1.52 |
|  |  |  |  |  |  |  |  |  |  |  |  |  |
| Between-Person (L-2) | SSS | **-0.07** | -0.14 | -0.01 |  | -0.03 | -0.11 | 0.04 |  | -0.04 | -0.08 | 0.01 |
|  | SSS*Quarter | 0.04 | -0.01 | 0.08 |  | -0.01 | -0.08 | 0.06 |  | -0.001 | -0.04 | 0.04 |
|  |  |  |  |  |  |  |  |  |  |  |  |  |
| Within-person (L-1) | SSS | 0.08 | -0.11 | 0.28 |  | 0.18 | -0.03 | 0.38 |  | **-0.14** | -0.27 | -0.02 |
|  | SSS*Quarter | -0.20 | -0.59 | 0.18 |  | -0.35 | -0.74 | 0.06 |  | 0.17 | -0.06 | 0.41 |
|  |  |  |  |  |  |  |  |  |  |  |  |  |

*Note:* The table reports results of sensitivity analyses removing sleep diaries received between 6:00 AM and 12:00PM the morning following the assigned diary day. Unstandardized Bayesian point estimates and 95% credible intervals around these estimates for each sleep outcome regressed by between-person SES predictors (L2) and within-person SES predictors (L1) are reported. Note that first-generation status contains only between-person variation. L-1 = Level-1; L-2 = Level-2; SSS = Subjective Social Status.

Supplemental Table 2. Multilevel associations of SES predictors with sleep variability after adjusting for the duration of participants’ sleep

|  |  | Sleep Variability | | |
| --- | --- | --- | --- | --- |
|  |  | Estimate | 2.5% | 97.5% |
|  |  |  |  |  |
| Between-Person (L-2) | First-Generation | **0.20** | 0.05 | 0.34 |
|  | First-Generation*Quarter | -0.04 | -0.18 | 0.10 |
|  |  |  |  |  |
|  | Sleep Duration | **-0.06** | -0.08 | -0.04 |
|  |  |  |  |  |
| Between-Person (L-2) | Financial Stress | **0.19** | 0.08 | 0.30 |
|  | Financial Stress*Quarter | 0.02 | -0.09 | 0.12 |
|  |  |  |  |  |
| Within-person (L-1) | Financial Stress | **-0.38** | -0.75 | -0.02 |
|  | Financial Stress*Quarter | **0.41** | -0.27 | 1.07 |
|  |  |  |  |  |
|  | Sleep Duration | **-0.06** | -0.08 | -0.04 |
|  |  |  |  |  |
| Between-Person (L-2) | SSS | -0.03 | -0.07 | 0.01 |
|  | SSS*Quarter | -0.01 | -0.05 | 0.03 |
|  |  |  |  |  |
| Within-person (L-1) | SSS | **-0.13** | -0.25 | -0.01 |
|  | SSS*Quarter | 0.14 | -0.08 | 0.37 |
|  |  |  |  |  |
|  | Sleep Duration | **-0.06** | -0.08 | -0.04 |

*Note:* The table reports results of sensitivity analyses wherein associations of SES predictors with sleep variability are adjusted for participants’ sleep duration. L-1 = Level-1; L-2 = Level-2; SSS = Subjective Social Status.

Supplemental Table 3. Multilevel associations of SES predictors with daily sleep outcomes on weekdays and weekends

|  |  |  | Difficulty Waking | | |  | Duration | | |  | Variability | | |
| --- | --- | --- | --- | --- | --- | --- | --- | --- | --- | --- | --- | --- | --- |
|  | Effect-Level | SES Index | *B* | 2.5% | 97.5% |  | *B* | 2.5% | 97.5% |  | *B* | 2.5% | 97.5% |
| **A. Weekdays** |  |  |  |  |  |  |  |  |  |  |  |  |  |
|  | Between-Person (L-2) | First-Generation | 0.06 | -0.20 | 0.34 |  | -0.21 | -0.49 | 0.08 |  | **0.20** | 0.04 | 0.36 |
|  |  | First-Generation*Quarter | -0.02 | -0.21 | 0.17 |  | 0.01 | -0.26 | 0.28 |  | -0.05 | -0.20 | 0.11 |
|  |  |  |  |  |  |  |  |  |  |  |  |  |  |
|  | Between-Person (L-2) | Financial Stress | **0.30** | 0.12 | 0.48 |  | -0.09 | -0.30 | 0.11 |  | **0.24** | 0.13 | 0.36 |
|  |  | Financial Stress*Quarter | 0.05 | -0.08 | 0.18 |  | -0.16 | -0.35 | 0.04 |  | -0.02 | -0.14 | 0.10 |
|  |  |  |  |  |  |  |  |  |  |  |  |  |  |
|  | Within-person (L-1) | Financial Stress | -0.23 | -0.91 | 0.51 |  | 0.67 | -0.04 | 1.39 |  | **-0.63** | -1.04 | -0.22 |
|  |  | Financial Stress*Quarter | 0.46 | -0.90 | 1.72 |  | -0.88 | -2.18 | 0.39 |  | 0.67 | -0.04 | 1.38 |
|  |  |  |  |  |  |  |  |  |  |  |  |  |  |
|  | Between-Person (L-2) | SSS | **-0.08** | -0.15 | -0.02 |  | 0.01 | -0.06 | 0.08 |  | -0.02 | -0.06 | 0.03 |
|  |  | SSS*Quarter | 0.02 | -0.03 | 0.06 |  | 0.02 | -0.05 | 0.09 |  | -0.03 | -0.07 | 0.01 |
|  |  |  |  |  |  |  |  |  |  |  |  |  |  |
|  | Within-person (L-1) | SSS | **0.27** | 0.06 | 0.49 |  | 0.22 | -0.01 | 0.44 |  | **-0.22** | -0.35 | -0.09 |
|  |  | SSS*Quarter | **-0.58** | -0.96 | -0.18 |  | -0.34 | -0.74 | 0.07 |  | **0.26** | 0.03 | 0.48 |
|  |  |  |  |  |  |  |  |  |  |  |  |  |  |
|  |  |  |  |  |  |  |  |  |  |  |  |  |  |
|  |  |  | Difficulty Waking | | |  | Duration | | |  | Variability | | |
|  | Effect-Level | SES Index | *B* | 2.5% | 97.5% |  | *B* | 2.5% | 97.5% |  | *B* | 2.5% | 97.5% |
| **B. Weekends** |  |  |  |  |  |  |  |  |  |  |  |  |  |
|  | Between-Person (L-2) | First-Generation | 0.16 | -0.14 | 0.47 |  | -0.09 | -0.51 | 0.33 |  | 0.18 | -0.05 | 0.41 |
|  |  | First-Generation*Quarter | -0.21 | -0.50 | 0.10 |  | 0.41 | -0.07 | 0.90 |  | -0.05 | -0.34 | 0.24 |
|  |  |  |  |  |  |  |  |  |  |  |  |  |  |
|  | Between-Person (L-2) | Financial Stress | **0.24** | 0.03 | 0.46 |  | 0.01 | -0.29 | 0.31 |  | 0.05 | -0.12 | 0.23 |
|  |  | Financial Stress*Quarter | 0.05 | -0.17 | 0.27 |  | 0.02 | -0.35 | 0.40 |  | 0.15 | -0.07 | 0.38 |
|  |  |  |  |  |  |  |  |  |  |  |  |  |  |
|  | Within-person (L-1) | Financial Stress | -0.44 | -1.24 | 0.37 |  | -0.06 | -1.20 | 1.05 |  | 0.13 | -0.47 | 0.74 |
|  |  | Financial Stress*Quarter | 0.62 | -0.78 | 1.96 |  | 0.08 | -1.73 | 2.01 |  | -0.04 | -1.04 | 0.96 |
|  |  |  |  |  |  |  |  |  |  |  |  |  |  |
|  | Between-Person (L-2) | SSS | -0.07 | -0.15 | 0.004 |  | -0.04 | -0.15 | 0.06 |  | -0.06 | -0.12 | 0.00 |
|  |  | SSS*Quarter | 0.05 | -0.03 | 0.12 |  | -0.03 | -0.16 | 0.09 |  | 0.04 | -0.04 | 0.11 |
|  |  |  |  |  |  |  |  |  |  |  |  |  |  |
|  | Within-person (L-1) | SSS | -0.09 | -0.35 | 0.19 |  | 0.22 | -0.15 | 0.57 |  | 0.03 | -0.17 | 0.22 |
|  |  | SSS*Quarter | 0.05 | -0.45 | 0.50 |  | -0.22 | -0.82 | 0.39 |  | -0.004 | -0.33 | 0.32 |
|  |  |  |  |  |  |  |  |  |  |  |  |  |  |

*Note:* The table reports results of sensitivity analyses evaluating associations of SES predictors with sleep outcomes separately for weekdays and weekends. L-1 = Level-1; L-2 = Level-2; SSS = Subjective Social Status.

Supplemental Equations for Multilevel Models of PSQI and sleep duration using Scott, Shroud & Weinberg (2013) Notation

1. Predicted PSQI at the i^th^ of three academic quarters (centered at fall) for the j^th^ student. CGM= centered at the grand mean. CWC=centered within-person cluster.

$${PSQI}_{ij}=\left( \beta_{0}+\mu_{0j} \right)+\beta_{1}\left( {FIRSTGEN}_{j} \right)+\beta_{2}\left( {QUARTER}_{ij} \right)+\beta_{3}\left( {QUARTER}_{ij} \right)\left( {FIRSTGEN}_{j} \right)+\beta_{4}\left( {GENDER}_{j} \right)+\beta_{5}\left( {ETHNICITY}_{j} \right)+\varepsilon_{ij}$$

$${PSQI}_{ij}=\left( \beta_{0}+\mu_{0j} \right)+\beta_{1}\left( cgm({FINSTRESS}_{j}) \right)+\beta_{2}\left( cwc({FINSTRESS}_{ij}) \right)+\beta_{3}\left( {QUARTER}_{ij} \right)+\beta_{4}\left( {QUARTER}_{ij} \right)\left( cgm({FINSTRESS}_{j}) \right)+\beta_{5}\left( {QUARTER}_{ij} \right)\left( cwc({FINSTRESS}_{ij}) \right)+\beta_{6}\left( {GENDER}_{j} \right)+\beta_{7}\left( {ETHNICITY}_{j} \right)+\varepsilon_{ij}$$

$${PSQI}_{ij}=\left( \beta_{0}+\mu_{0j} \right)+\beta_{1}\left( cgm({SSS}_{j}) \right)+\beta_{2}\left( cwc({SSS}_{ij}) \right)+\beta_{3}\left( {QUARTER}_{ij} \right)+\beta_{4}\left( {QUARTER}_{ij} \right)\left( cgm({SSS}_{j}) \right)+\beta_{5}\left( {QUARTER}_{ij} \right)\left( cwc({SSS}_{ij}) \right)+\beta_{6}\left( {GENDER}_{j} \right)+\beta_{7}\left( {ETHNICITY}_{j} \right)+\varepsilon_{ij}$$

1. Predicted sleep diary outcome on the i^th^ of 28 diary days (centered at the fall academic quarter) for the j^th^ student. CGM= centered at the grand mean. CWC=centered within-person cluster. Models for sleep variability and difficulty waking have similar expressions.

$${Duration}_{ij}=\left( \beta_{0}+\mu_{0j} \right)+\beta_{1}\left( {FIRSTGEN}_{j} \right)+\beta_{2}\left( {QUARTER}_{ij} \right)+\beta_{3}\left( {QUARTER}_{ij} \right)\left( {FIRSTGEN}_{j} \right)+\beta_{4}\left( {GENDER}_{j} \right)+\beta_{5}\left( {ETHNICITY}_{j} \right)+\varepsilon_{ij}$$

$${Duration}_{ij}=\left( \beta_{0}+\mu_{0j} \right)+\beta_{1}\left( cgm({FINSTRESS}_{j}) \right)+\beta_{2}\left( cwc({FINSTRESS}_{ij}) \right)+\beta_{3}\left( {QUARTER}_{ij} \right)+\beta_{4}\left( {QUARTER}_{ij} \right)\left( cgm({FINSTRESS}_{j}) \right)+\beta_{5}\left( {QUARTER}_{ij} \right)\left( cwc({FINSTRESS}_{ij}) \right)+\beta_{6}\left( {GENDER}_{j} \right)+\beta_{7}\left( {ETHNICITY}_{j} \right) {+ \varepsilon}_{ij}$$

$${Duration}_{ij}=\left( \beta_{0}+\mu_{0j} \right)+\beta_{1}\left( cgm({SSS}_{j}) \right)+\beta_{2}\left( cwc({SSS}_{ij}) \right)+\beta_{3}\left( {QUARTER}_{ij} \right)+\beta_{4}\left( {QUARTER}_{ij} \right)\left( cgm({SSS}_{j}) \right)+\beta_{5}\left( {QUARTER}_{ij} \right)\left( cwc({SSS}_{ij}) \right)+\beta_{6}\left( {GENDER}_{j} \right)+\beta_{7}\left( {ETHNICITY}_{j} \right)+\varepsilon_{ij}$$
